# Comparison of scleral buckling and vitrectomy using wide angle viewing system for rhegmatogenous retinal detachment in patients older than 35 years

**DOI:** 10.1186/s12886-015-0109-9

**Published:** 2015-09-11

**Authors:** Sung Who Park, Han Jo Kwon, Ho Yun Kim, Ik Soo Byon, Ji Eun Lee, Boo Sup Oum

**Affiliations:** Department of Ophthalmology, Pusan National University Hospital, Pusan, South Korea; Department of Ophthalmology, Pusan National University Yangsan Hospital, Yangsan, South Korea; Medical Institute, School of Medicine, Pusan National University, Pusan, South Korea; Biomedical Research Institute, Pusan National University Hospital, Pusan, South Korea

## Abstract

**Background:**

To compare scleral buckling (SB) and pars plana vitrectomy (PPV) using a wide angle viewing system (WAVS) for uncomplicated phakic rhegmatogenous retinal detachment (RRD).

**Methods:**

The medical records of patients with uncomplicated phakic RRD were retrospectively reviewed. Eyes with pseudophakic or attached fovea were excluded. Patients treated with SB were classified as group B, and PPV using WAVS as group V. Primary success rate, visual acuity (VA), macular complications, and sustained subretinal fluid (SRF) were compared between groups.

**Results:**

Seventy-two eyes were included in group B and 57 eyes in group V. Group B had better preoperative VA (1.38 ± 0.87 vs 1.84 ± 0.97 in LogMAR, *P* = 0.010), but worse final VA (0.51 ± 0.48 vs 0.30 ± 0.23, *P* = 0.012) than group V. The primary success rate of 94.7 % in group V was higher than 77.8 % in group B (*P* = 0.010). Final success rate was 100 % in both groups. There was no significant difference in macular complications between groups (*P* = 0.087). Sustained SRF was found in 22 eyes in group B (38.6 %), while only two eyes in group V exhibited sustained SRF (2.8 %, *P* < 0.001).

**Conclusions:**

Pars plana vitrectomy using WAVS was more efficacious than SB for treating uncomplicated phakic RRD.

## Background

Rhegmatogenous retinal detachment (RRD), which refers to detachment of the sensory retina from the retinal pigment epithelium caused by breaks in the retina, is an important cause of permanent visual loss [1]. Scleral buckling (SB) and pars plana vitrectomy (PPV) are the two major surgical treatments for RRD.

PPV was reported to be more efficacious in pseudophakic eyes [2–4], and many surgeons prefer SB in younger phakic RRD without posterior vitreous detachment (PVD) and those with less-liquefied, formed vitreous [5, 6]. Previous randomized clinical trials have showed comparable efficacy between the two surgical procedures for treating uncomplicated phakic RRD [2, 5, 7, 8]. Procedure choice is determined as per the surgeon’s discretion in uncomplicated phakic RRD.

The safety and effectiveness of PPV has been improving owing to more technically advanced surgical instruments including a wide angle viewing system (WAVS) [9]. The WAVS enhances surgical procedures, especially for retinal detachment, because it provides comprehensive information regarding the configuration of the retinal detachment and an excellent surgical view after fluid-air exchange. Thus, the efficacy of vitrectomy for retinal detachment should be reappraised in the WAVS era.

The aim of the present study is to compare the surgical outcomes of SB and PPV using WAVS in patients with uncomplicated phakic RRD.

## Methods

### Design

Retrospective comparative analysis of an interventional case series.

### Participants

129 eyes treated with SB or PPV with WAVS for RRD were included.

The Ethical Committee of Pusan National University Hospital approved this study in accordance with the rules set forth in the Helsinki Declaration. A retrospective review was performed on the medical records of patients who underwent either SB or PPV for RRD and were followed up at least 3 months in Pusan National University Hospital from Jan. 2011 to Sep. 2013.

Patients younger than 35 years were excluded from the present study because the primary procedure in our hospital is SB for uncomplicated RRD in patients younger than 35 year-old, as our database indicates that they have a high success rate. Moreover, vitrectomy for younger patients may cause postoperative cataract progression and subsequent loss of accommodation after cataract surgery. In addition, an epidemiologic study revealed two peaks in the incidence of RRD [10], suggesting an alternative mechanism underlying the cause of PVD in younger patients [11].

RRD complicated with severe media opacity, proliferative vitreoretinopathy grade C, posterior retinal break, or pseudophakia were defined as complicated and excluded. If the patients underwent combined PPV with SB, or had attached fovea or other ocular disease impacting visual acuity (VA), they were also excluded.

Patients treated with SB were classified as group B, while vitrectomy with WAVS was classified as group V. SB was combined with cryoretinopexy in all cases. Subretinal fluid (SRF) was drained and/or gas was injected into the vitreous cavity at each surgeon’s discretion. PPV was performed using Accurus (Alcon, Fort Worth, TX, USA). The fundus was visualized using WAVS: BIOM (Oculus, Wetzlar, Germany) or Resight 700 (Carl Zeiss Meditec AG, Jena, Germany). The 23- or 25-gauge cutters were used at a rate of 2,500–5,000 cuts per minute. Cataract surgery was performed concurrently to prevent postoperative cataract progression with patient consent. If necessary to confirm the presence of PVD or epiretinal membrane (ERM), triamcinolone acetonide was applied during the PPV. For shaving peripheral vitreous, an assistant indented the sclera.

Prophylactic photocoagulation was applied only around retinal breaks or lesions predisposed to retinal detachment, not on normal looking retina. Sulfur hexafluoride (SF_6_), octafluoropropane (C_3_F_8_), room air, or silicone oil were used as tamponade at the surgeon’s discretion.

The baseline characteristics evaluated included age, preoperative VA, detachment area, symptoms duration, number of breaks, intraocular pressure, presence of a tear larger than 0.5 disc diameters, and presence of PVD. Detachment area was measured as clock hours at the equator. Presence of PVD was evaluated in group V during PPV.

Primary success was defined as the retina maintaining reattached for at least 3 months after the primary surgery. Localized small SRF without an increase during follow-up was not considered surgical failure. Macular complication was defined as full-thickness macular hole or ERM that required surgical intervention. Sustained submacular fluid (SMF) was defined as SRF persisting in the macula detected using spectral-domain optical coherence tomography (OCT) at 3 months or later.

Primary success rate, VA, macular complication, operation time and sustained SMF were compared between the two groups. VA, age, symptom duration, number of breaks, detachment area, intraocular pressure, operation time, and follow-up duration were compared using the Mann–Whitney *U* test, and categorical variables, including sex, presence of tear, and primary success, macular complication, and sustained SMF using Chi-square test or Fisher’s exact test. Statistical analyses were performed using IBM SPSS Statistics 21, (IBM Inc., Armonk, NY, USA) setting the level of statistical significance at *P* < 0.05.

## Results

A total of 129 eyes were included in the study; 72 eyes in group B and 57 eyes in group V. Four surgeons performed the operations, and no significant differences were found between their preferences or success rates. Baseline characteristics of each group are summarized in Table 1. There was no significant difference in age, sex, detachment area, intraocular pressure, or presence of a large tear between the two groups. The number of breaks was 1.5 ± 0.9 in group B, which was less than 1.9 ± 1.2 in group V (*P* = 0.013). In only one eye (1.8 %) in group V, the presence of vitreo-papillary adhesion was noticed during vitrectomy.

**Table 1:** The demographics and clinical data. Values are presented as mean ± standard deviation

	Group B	Group V	*P*-value
Number	72	57	
Age (Years)	54.4 ± 11.7	56.3 ± 9.6	0.178^*^
Sex (Male/Female)	44/28	34/23	1.000^**^
Symptom duration (Days)	21.8 ± 46.7	18.8 ± 37.2	0.978^*^
Posterior vitreous detachment (%)	NA^†^	98.2 %	NA^†^
The number of tear (*N*)	1.5 ± 0.9	1.9 ± 1.2	0.013^*^
Preoperative intraocular pressure (mmHg)	11.5 ± 2.5	11.1 ± 3.9	0.914
Presence of large tear (*N*, %)	58 (80.6 %)	52 (91.2 %)	0.133^***^
Initial visual acuity (LogMAR)	1.38 ± 0.87	1.84 ± 0.97	0.010^*^
Detachment area (hours)	6.1 ± 2.7	5.6 ± 2.2	0.186^*^
Follow up (Months)	6.9 ± 5.2	6.6 ± 5.1	0.409^*^

*Mann–Whitney *U* test

**Chi square test

***: Fisher’s exact test

^†^Not applicable

In group B, encircle, circumferential segmental buckle, and radial buckle were performed in 9 eyes (12.5 %), 62 eyes (86.1 %) and 1 eye (1.4 %), respectively. SRF was drained in 65 eyes (90.3 %). Intravitreal gas was injected in 10 eyes (13.9 %) during the operation. In group V, 46 eyes (80.7 %) underwent cataract operation concurrently. Retinotomy was performed to drain SRF in two eyes (3.5 %). SF_6_, C_3_F_8_, room air, and silicone oil were used as tamponade in 36 (63.2 %), 14 (24.6 %), 3 (5.3 %), and 4 (7.0 %) eyes, respectively. And silicone oil was removed by 3 months after operation.

**Fig. 1:**
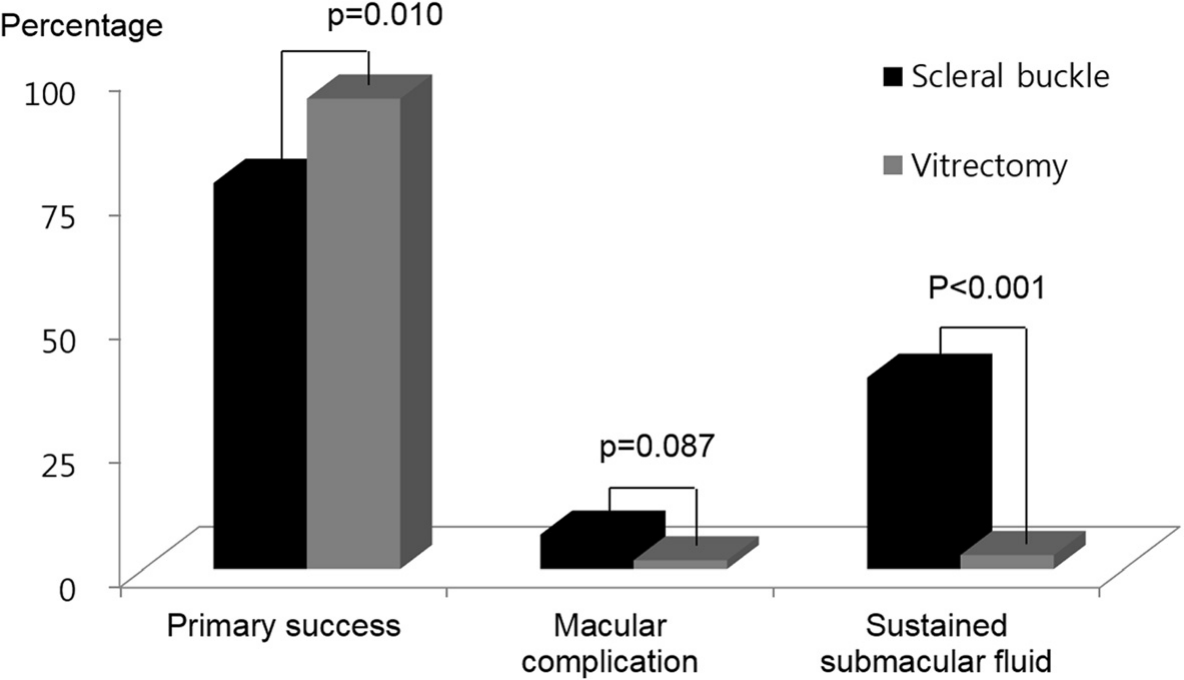
Anatomical outcomes after scleral buckling versus vitrectomy using wide angle viewing system for rhegmatogenous retinal detachment. Vitrectomy achieved more frequent primary success and less frequent sustained submacular fluid (Fisher’s exact test)

**Fig. 2:**
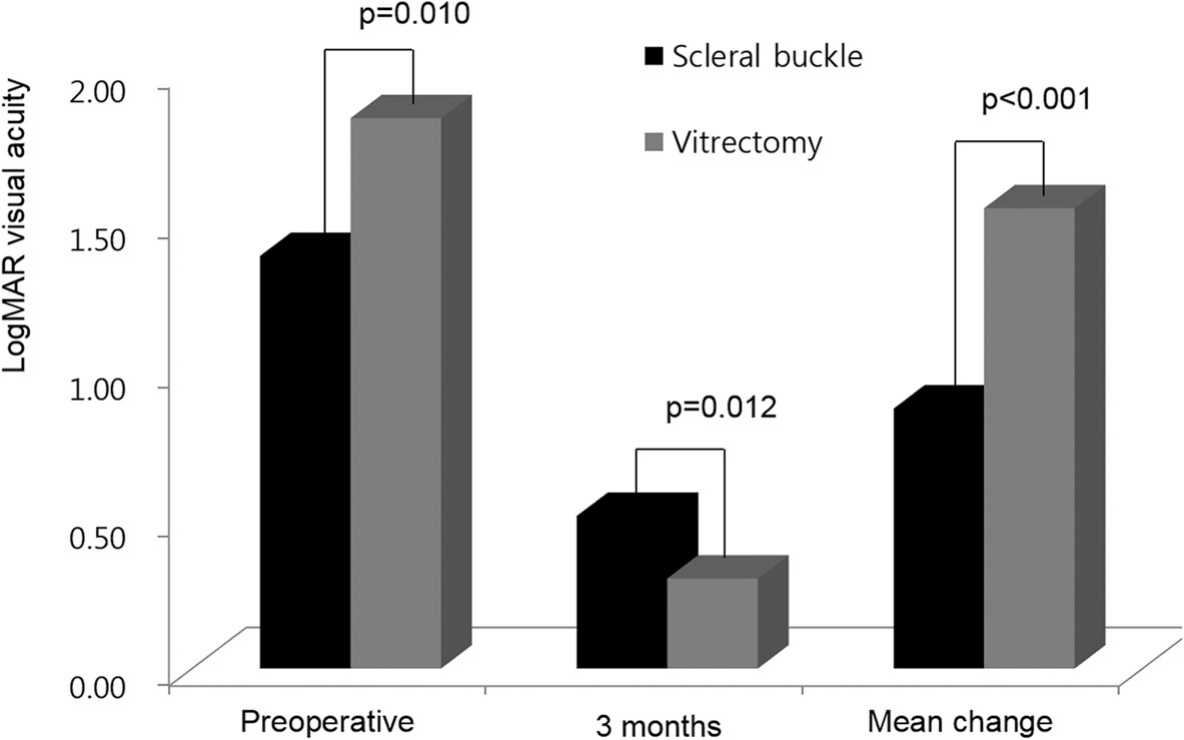
Visual acuity before and after surgery between scleral buckling and vitrectomy the using wide angle viewing system

Primary success was achieved in 54 eyes (94.7 %) in group V, which was significantly higher than 56 eyes (77.8 %) in group B (*P* = 0.010, Fig. 1). Additional procedures, including photocoagulation or intravitreal gas injection, were performed in 17 (23.6 %) eyes in group B, which were more frequent than 3 (5.3 %) eyes in group V (*P* = 0.009). Final success rate was 100 % in both groups. Preoperative VA (LogMAR) in group B (1.38 ± 0.87) was better than in group V (1.84 ± 0.97, *P* = 0.010), whereas visual acuity at 3 months after surgery in group B (0.51 ± 0.48) was worse than in group V (0.30 ± 0.23, *P* = 0.012, Fig. 2). Visual improvement in group V (1.53 ± 0.93) was significantly greater than 0.88 ± 1.00 in group B (*P* < 0.001, Fig. 2).

Three cases of macular hole and two cases of ERM required a second operation in group B, and one case of ERM in group V (*P* = 0.087, Fig. 1). Sustained SMF was found in 22 eyes in group B (38.6 %), which was more common than two eyes in group V (2.8 %; *P* < 0.001, Fig. 1). Operation time of group B was 63.0 ± 39.2 min and group V was 60.8 ± 15.2. There was no significant difference of operation time (p = 0.246). Two cases of re-detachment were noticed after 3 months. One was noticed at 9 months after SB and the other one occurred immediately after ocular trauma at 6 months after PPV.

## Discussion

The present study compared two surgical methods retrospectively for treating uncomplicated phakic RRD; PPV using WAVS was more efficacious than SB in terms of functional outcome as well as primary anatomical success.

For several decades, even after the advent of PPV in 1971 by Machemer et al. [12], SB had been the standard treatment for RRD. In recent years, evolution of surgical instruments has made PPV competitive with SB [13], and occasionally superior to SB in specific situations, such a patients presenting with poor SB prognostic factors, such as pseudophakia [2, 14], media opacity, and proliferative vitreoretinopathy [6, 15–17].

Although many studies have been conducted to compare the efficacy of the two procedures prospectively or retrospectively, there is still no consensus on the optimal approach for the management of uncomplicated phakic RRD [2, 5, 7, 8, 18, 19]. There were a few comparative data of two methods in uncomplicated phakic RRD. In 2001, Oshima et al. [18] and Miki et al. [5] compared retrospectively. In 2007 there were 3 prospective comparative studies. Koriyama et al. [7] and Azad et al. [8] evaluated in small size population, while Heimann et al. [2] conducted multicenter randomized clinical trials. In 2013, Adelman et al. [19] reported a retrospective comparative study of over 7,000 patients in 48 countries. But, they didn't show any superiority in terms of primary anatomical success.

Despite a high success rate of over 95 % with PPV [6, 20] and its growing popularity, Heimann et al. [21] pointed out that this trend has not been justified by clinical results.

The superior outcome following PPV in the current study may be explained by a few characteristics that differ from previous studies. First, WAVS was used with all cases of PPV. Although not specifically mentioned, the previous studies seemed to be composed of non-WAVS or both systems. WAVS is a key advancement in vitrectomy for RRD because it enables visualization of the configuration of detachment, visualization of peripheral breaks and the macula simultaneously, and provides excellent view after fluid-gas exchange. In addition, WAVS provides a wider and better view under the presence of media opacity or small pupils [22].

Second, patients younger than 35 years old were excluded in the present study as RRD in these patients follows a different mechanism other than PVD [10]. Young patients usually have less-liquefied vitreous, and SB has a high success rate. In addition, SB may avoid cataract after vitrectomy. On the other hand, PPV in young phakic patients would be less practicable than older patients in several aspects: PVD induction and extension, and removing the peripheral vitreous.

Considering the above, the higher anatomical success rate of PPV is related to vitreous liquefaction and the presence of PVD. PVD was confirmed in 98.2 % of patients during PPV the current study. More liquefied vitreous with PVD may play a role as counter action the buckle effect; this type of vitreo-retinal traction can be removed more completely by PPV than SB.

On the other hands, visual recovery was better after PPV than SB. In addition to the higher primary success rate, less frequent sustained SMF and combined cataract surgery might be the reason of better visual outcomes. Sustained SMF is observed more frequent after SB and is related to delayed visual recovery [23]. PPV is more advantageous to drain SRF completely and to achieve early reattachment.

There are several weaknesses to our study. Due to the retrospective nature of this study, patients were not randomized, and there were discrepancies in some baseline characteristics. Low initial VA and multiple breaks in group V implied the surgeons’ preference for PPV in eyes with bullous RD. As all of these factors are related to poor prognosis [24, 25], these discrepancies might actually strengthen our results showing the superiority of PPV over SB. The other weaknesses include the short follow-up period and the small number of patients from a single center. Furthermore, 3 months of follow-up as inclusion criteria is relatively short to assess final visual outcome. Nevertheless, it is sufficient to assess the primary success rate and might reduce selection bias.

## Conclusions

PPV is known as better option for complicated RRD, which is combined with pseudophakia [2–4], posterior retinal break or proliferative vitreoretinopathy C. The current study showed that PPV using WAVS is more efficacious for treating not only complicated RRD but also uncomplicated than SB with respect to both functional and anatomical outcomes. Our results should be confirmed by a large scale, prospective, randomized clinical trial.

## Abbreviations

(SB): Scleral buckling
(PPV): Pars plana vitrectomy
(WAVS): Wide angle viewing system
(RRD): Rhegmatogenous retinal detachment
(VA): Visual acuity
(SRF): Subretinal fluid
(PVD): Posterior vitreous detachment
(SF_6_): Sulfur hexafluoride
(C_3_F_8_): Octafluoropropane
(ERM): Epiretinal membrane
(SMF): Submacular fluid
(OCT): Optical coherence tomography
